# Red ginseng extract improves skeletal muscle energy metabolism and mitochondrial function in chronic fatigue mice

**DOI:** 10.3389/fphar.2022.1077249

**Published:** 2022-12-23

**Authors:** Haijing Zhang, Chunhui Zhao, Jinli Hou, Ping Su, Yifei Yang, Bing Xia, Xiaoang Zhao, Rong He, Lifang Wang, Chunyu Cao, Ting Liu, Jixiang Tian

**Affiliations:** ^1^ Institute of Chinese Materia Medica, China Academy of Chinese Medical Sciences, Beijing, China; ^2^ Experimental Research Center, China Academy of Chinese Medical Sciences, Beijing, China

**Keywords:** red ginseng, energy metabolism, chronic fatigue, mitocchondrial dysfunction, AMPK

## Abstract

**Background:** Skeletal muscles are organs with high energy requirements, especially during vigorous exercise. Adequate mitochondrial function is essential to meet the high energy needs of skeletal muscle cells. Recent studies have reported that red ginseng can significantly improve chronic fatigue; however, the specific mechanism of action is still not clear.

**Methods:** A chronic fatigue syndrome mouse model was developed using C57BL/6J mice through long-term compound stimulation of stress factors. Following this, the animals were orally administered 200, 400, or 600 mg/kg red ginseng extracts for 28 days. Skeletal muscle lactate acid, serum lactate dehydrogenase, urea concentrations, ATP level, mitochondrial membrane potential, activities of Na^+^-K^+^-ATPase and cytochrome c oxidase were determined using assay kits or an automatic biochemical analyser detection system. Skeletal muscle mitochondria morphology was observed using electron microscopy and the expression of p-AMPK, PGC-1α, ACO2 and complex I in skeletal muscle protein was determined by western blotting.

**Results:** Oral administration of 400 or 600 mg/kg red ginseng extract in mice with chronic fatigue reduced lactic acid, lactate dehydrogenase and urea, rescued the density and morphology of skeletal muscle mitochondria, increased the activities of Na^+^-K^+^-ATPase and cytochrome c oxidase, and activated the AMPK/PGC-1α cascade pathway, resulting in improved skeletal muscle mitochondrial function by restoring ATP level, mitochondrial membrane potential, complex I and mitochondrial biogenesis.

**Conclusion:** The anti-fatigue effects of red ginseng are partly related to its potent mitochondrial improving activity, including decreasing mitochondrial swelling and mitochondrial membrane permeability, increasing mitochondrial biogenesis, thus ameliorating mitochondrial dysfunction.

## 1 Introduction

Chronic fatigue is commonly defined as a complicated physiological phenomenon that results in the physiology of the body being unable to maintain its function at a normal level and/or one or more organs failing to sustain regular intensity after exercise (Anderson and Maes, 2020; Hou et al., 2020). Chronic fatigue does not only reduce the exercise ability of the body, but also results in the body developing associated physiological, biochemical, and pathological changes (Natelson et al., 2021). Reducing or alleviating the decline in exercise ability caused by chronic fatigue has always been a joint research topic of sport and medicine related disciplines (Sandler and Lloyd, 2020). To this end, numerous herbal medicines has been extensively used satisfactorily to improve exercise ability through its anti-fatigue properties (Liu et al., 2021; Panossian et al., 2021).

Red ginseng (*Ginseng Radix et Rhizoma Rubra*) is processed by steaming and drying of the root and rhizome of Araliaceae plant Ginseng (*Panax ginseng* C.A. Meyer). It is warm in nature, sweet and slightly bitter in taste, with the effects of tonifying vital energy, relieving pulse, invigorating Qi and absorbing blood (Zhao et al., 2018). It is used for treating various conditions including Qi deficiency, limb cold, heart failure, and cardiogenic shock (Park et al., 2021; Yoon et al., 2021). Numerous studies have shown that ginseng is rich in a variety of ingredients (Lee et al., 2015). Owing to this, diverse components are present in red ginseng, with this being attributed to a series of changes that take place because of the steaming process (Wei-Xu et al., 2021). According to the literature, the chemical constituents of red ginseng comprise of triterpenoid saponins, volatile oils, polysaccharides, amino acids, and trace elements, all of which contribute to various pharmacological effects including enhancing immunity, preventing fatigue, strengthening physique, anti-oxidation, and anti-aging (So et al., 2018; Hyun et al., 2020; Yoon et al., 2021). As one of the vital processed products of *P. ginseng*, red ginseng has been used as a health-promoting supplement to improve stamina and vitality in clinical settings globally for centuries (Lee et al., 2015).

As the energy factory of cells, mitochondria are closely related to energy metabolism. Adenosine 5‘-monophosphate—activated protein kinase (AMPK)/peroxisome proliferator-activated receptor-γ coactivator-1α (PGC-1α) is a pathway of intracellular energy metabolism that plays a vital role in promoting mitochondrial fission and fusion, as well as improving energy metabolism. AMPK can up-regulate the downstream target molecule PGC-1α to maintain the stability of energy metabolism (McConell et al., 2010). A recent study showed that the water extract of red ginseng prolonged the weight-bearing swimming time of fatigue mice, reduced the concentration of serum lactic acid, enhanced the adaptability of the body to exercise load through antioxidant activity, and played an anti-fatigue role (Zhang and Guo, 2020).

Herein, we investigated the effect of long-term exposure to multiple stress factors in stimulating the AMPK/PGC-1α pathway in mouse skeletal muscle by first establishing a mouse model of chronic fatigue syndrome (CFS). Next, we determined the effect of red ginseng against chronic fatigue and its associated mechanism of action. This work seeks to provide a scientific basis for the use of red ginseng in treating body fatigue, and thus providing a new platform for the clinical development of anti-fatigue drugs.

## 2 Materials and methods

### 2.1 Materials

The red ginseng extract (RGE) was manufactured and provided by Jilin Hanzheng Ginseng Co., Ltd (Lot Number: N60221006; Yanbian Korean Autonomous Prefecture, Jilin Province, China) according to the “Enterprise Standard of Red Ginseng Extract” filed with Health Commission of Jilin Province. Generally, red ginseng was made from fresh root of five-years-old ginseng (*Panax ginseng* C.A.Meyer, Jilin Changbaishan, Jilin, China) after washing, steaming (50°C–98°C, 4 h) and drying (60–70°C, 15 h) process; and then, the red ginsengs were extracted with water (87°C, 4 times for 12 h), followed by concentrating, sterilizing, filtrating process to produce RGE. 100 g concentrated fluid was extracted and concentrated from 140 g five-years-old red ginseng. The RGE contains more than 60% (W/W) of soluble solid content and more than 3% (W/W) of total ginsenoside. The calculated content of total ginsenoside were 35.2 mg per Gram of RGE using ultraviolet spectrophotometry. The separation pattern of ginsenosides in RGE was presented in Supplementary Figure S1 using HPLC-photo diode array analysis. As prescribed in “Announcement on Approval of Ginseng (Artificial Cultivation) as a New Resource Food (Ministry of Health Announcement No. 17 of 2012, People’s Republic of China)”, the suggested dosage for adults is no more than 3 g/day (crude drug), which converted to the equivalent dosage for mouse is about 530 mg/kg/day of crude drug, or 380 mg/kg/day of RGE. Thus, we applied 200, 400 and 600 mg/kg/day of RGE in the current study.

### 2.2 Animals and experimental design

Experimental C57BL/6J mice (body weight: 18.0–21.0 g) were purchased from Vital River Laboratory Animal Technology Co., Ltd. (Beijing, China). All animal procedures used in this study were approved by the Institute of Chinese Materia Medica, China Academy of Chinese Medical Sciences Animal Care & Welfare Committee. The mice were sacrificed by cervical dislocation after being anesthetized. All efforts were made to minimize the number of animals used and their suffering. Five mice were housed in each cage under controlled photoperiods (12:12-h light–dark cycle) and temperature (23°C ± 1°C) and given with food and water *ad libitum*. Before the experiment, the mice were housed under these conditions for 2–3 days to adapt to the environment.

Chronic fatigue syndrome (CFS) model was established by applying multiple stress factors stimulation as described previously (Yin et al., 2021), with slight modifications. Briefly, mice were subjected to different forced stimuli for 14 consecutive days, including rota-rod test (15 rpm) for 13 min, cold water swimming (10°C ± 1°C) for 9 min, and sleep deprivation. Mice were applied cold water swimming and sleep deprivation on odd days, while rota-rod test (RRT) and sleep deprivation on even days. After treatment, the CFS mice present decreased activity, duller fur and loose stool. In addition, endurance capacity was assessed by subjecting the CFS mice to weight-loaded forced swimming test (WLFST). The mice in control group were kept in the same rearing environment but receiving no stimuli for 14 days.

At the 15th day, CFS mice were randomly divided into four groups (n = 13/group) and treated with pure water (CFS model group) or RGE (200, 400, 600 mg/kg) *via* intragastric administration once per day for 28 days. The CFS mice in model group and treatment group received different stimuli (cold water swimming/sleep deprivation or rota-rod/sleep deprivation) alternately from the 15th day to the 43rd day every other day. After evaluate the endurance capacity at the 44th day and 46th day, all of the mice were sacrificed and tissues were harvested immediately after last evaluation.

### 2.3 Evaluate the endurance capacity

At the 44th day and 46th day, the swimming endurance capacity of all mice in each group was evaluated blindly using WLFST and RRT.

Weight-loaded forced swimming test. At the 44th day, 1 h after the administration of RGE, all mice underwent WLFST with a tin wire (corresponding to 5% of their body weight) attached to tail base of the mouse. Each mouse was dropped individually into a swimming tank (25°C ± 1°C, 20 cm in diameter, 25 cm in depth). Exhaustion was determined by failed to return to the surface and keep their nose out of water within 5 s, the exhausted swimming time was recorded immediately (Liu et al., 2018).

Rota-rod test. At the 46th day, 1 h after the administration of RGE, all mice were placed on a rotating rod (Jinan Yiyan Science Technology Co., Ltd, Jinan, China) with a 3 cm diameter and 15 rpm rotation speed until they were exhausted and falling off from the rotating rod. The latency for each mouse before dropped was recorded (Zhu et al., 2021).

### 2.4 Determination of lactic acid (skeletal muscle), lactic acid dehydration (serum) and urea (serum)

Tibialis anterior (TA) muscles of mice were homogenized in PBS buffer. The lactate acid (LA) of TA muscle homogenates were determined using assay kits from the Nanjing Jiancheng Bioengineering Institute (Nanjing, China) according to the manufacturer’s instructions, and the absorbance value was measured on a microplate reader (BioTek Synergy H1, Vermont, United States) using wavelength of 570 nm.

Blood samples from each mouse were obtained from abdominal aorta after anesthetized, and centrifuged at 1 200 g and 4°C for 15 min to separate serum following the endurance test. Serum lactic acid dehydration (LDH) and urea were analyzed by Toshiba TBA-120FR automatic biochemical analyzer detection system using assay kits of LDH and urea (Autobio, Beijing, China).

### 2.5 Observation on the morphology of skeletal muscle mitochondria

After the endurance test, the morphology of mitochondria in mice skeletal muscle was observed by electron microscopy. After anesthesia, the right lower limb was taken about 0.5 mm × 1 mm × 3 mm long strip of TA muscle tissue. After fixed in 2.5% glutaraldehyde solution for 48 h, muscle tissues were dehydrated, embedded and sliced. They were stained with 3% uranium acetate lead citrate for 10 min. The ultra-structural changes of skeletal muscle mitochondria were observed by transmission electron microscopy Jem-1200EX (JEOL, Akishima, Japan). Mitochondrial area and aspect ratio were measured and quantified using the ImageJ software (National Institutes of Health, MD, United States).

### 2.6 Mitochondrial DNA quantification

Total DNA was extracted from TA muscle tissue using DNeasy blood and tissue kit (QIAGEN, Hilden, Germany) according to the manufacturer’s protocol. The relative expression of NADH dehydrogenase 1 (mtDNA) to TFAM (nuclear DNA) was quantified using real-time quantitative PCR to determine the relative copy number differences of mitochondrial DNA (mtDNA). The primers used in this study:

mtDNA forward:5′-CCTATCACCCTTGCCATCAT-3′;

mtDNA reverse: 5′-GAG​GCT​GTT​GCT​TGT​GTG​AC-3′;

Nuclear DNA forward: 5′-CTG​CAC​TCT​GCC​CAT​CCA​AA-3′;

Nuclear DNA reverse: 5′-CTG​AGC​ATT​CGC​AGG​CCT​TT-3′.

### 2.7 Western blot analysis

The same part of TA muscle tissues obtained from each mouse were homogenized and lysed with lysis buffer (RIPA buffer with complete protease inhibitors and protein phosphatase inhibitors) for 15 min, and then centrifuged at 10,000 rpm for 10 min at 4°C. The total protein concentration in supernatant was determined by the bicinchoninic acid method (Merck Millipore, United States). Equal amounts of protein were separated *via* 12% SDS polyacrylamide gel electrophoresis (Bio-Rad, United States) and transferred to nitrocellulose membranes. After blocking with 5% bovine serum albumin (BSA) for 2 h, the membranes were incubated with the following primary antibodies overnight at 4°C: rabbit monoclonal to phospho-AMPK (1:500; catalog 2535, Cell Signaling Technology, United States), total-AMPK (1:500; catalog 2603, CST), ACO2 (1:1000; catalog 6571T, CST), rabbit polyclonal to PGC1α (1:800; catalog ab54481, Abcam, United States), mouse monoclonal to NDUFB8 (1:1000; catalog 459210, Abcam), GAPDH (1:2000; catalog 97166, CST). The membranes were rinsed with 0.01 M phosphate-buffered saline (PBS) plus 0.1% Tween-20 and then incubated with horseradish peroxidase-conjugated goat anti-rabbit (sc-2004, Santa Cruz, United States) or goat anti-mouse (sc-2005, Santa Cruz, United States) secondary antibody for 2 h at room temperature. After that, the membranes were visualized with an electrochemiluminescence detection reagents (Beyotime, Shanghai, China) and captured using Amersham Imager 680 (GE Healthcare, California, United States). Band intensity was quantified using the ImageJ software (National Institutes of Health, MD, United States).

### 2.8 Determination of skeletal muscle Na^+^-K^+^-ATPase activity, cytochrome c oxidase (CcO) and ATP level

Na^+^-K^+^-ATPase activity, cytochrome c oxidase and ATP level were determined according to the manufacturer’s protocol. Briefly, the fresh TA muscles dissected from the left lower limb were homogenized using High-throughput Tissue Grinder (SCIENTZ-48, Scientz Biotechnology, Ningbo, China). The Na^+^-K^+^-ATPase activity, CcO activity and ATP level of homogenates were measured using Na^+^-K^+^-ATPase assay kit, cytochrome C oxidase assay kit or ATP assay kit (Nanjing Jiancheng Bioengineering Institute) by microplate reader (BioTek Synergy H1, United States).

### 2.9 Determination of skeletal muscle mitochondrial ROS and mitochondrial membrane potential

To verify the effect of RGE on CFS-induced oxidative stress, we determined intracellular reactive oxygen species (ROS) levels by separating and extracting the mitochondria from TA muscle, followed by measuring the fluorescence intensity of dichlorofluorescein (DCF) in mitochondria. TA muscle mitochondria were isolated using Mitochondrial Fractionation Kit (Active Motif, Carlsbad, CA, United States) and quantified using BCA protein assay kit. The isolated mitochondria were incubated with 10 μM 2′,7′-dichlorodihydrofluorescein diacetate (DCFH-DA) for 20 min at 37°C. The fluorescence intensity of DCF was captured and analyzed by a fluorescence microplate reader (BioTek Synergy H1, United States) with excitation at 485 nm and emission at 528 nm. TA muscle mitochondrial ROS levels were presented as arbitrary units (a.u.).

To examine whether RGE could preserve mitochondrial function, we assessed mitochondrial membrane potential (△ΨM) using JC-1 mitochondrial transmembrane potential detection kit (Cell Technology, Fremont, United States). The isolated TA muscle mitochondria were stained with 0.5 ml of 1× JC-1 reagent and incubated at 37°C in a 5% CO_2_ incubator for 20 min. Then the mitochondrial membrane potentials were detected using fluorescence microplate with Ex485 nm (Em535 nm) and Ex550 nm (Em600 nm). The mitochondrial membrane potential was presented as the ratio of JC-1 red to green fluorescence (Em600 nm/Em535 nm).

### 2.10 Statistical analysis

All data were analyzed using SPSS 20.0 software (IBM, Armonk, NY) and GraphPad Prism 8.0 software (San Diego, CA, United States), expressed as the mean ± standard error of the mean (SEM). For normally distributed data, statistical analysis was conducted with one-way ANOVA, followed by Fisher’s protected least significant difference test for *post hoc* comparisons. *p* < 0.05 was considered significant.

## 3 Results

### 3.1 Red ginseng ameliorates multiple stress factors-induced chronic fatigue in mice

**FIGURE 1:**
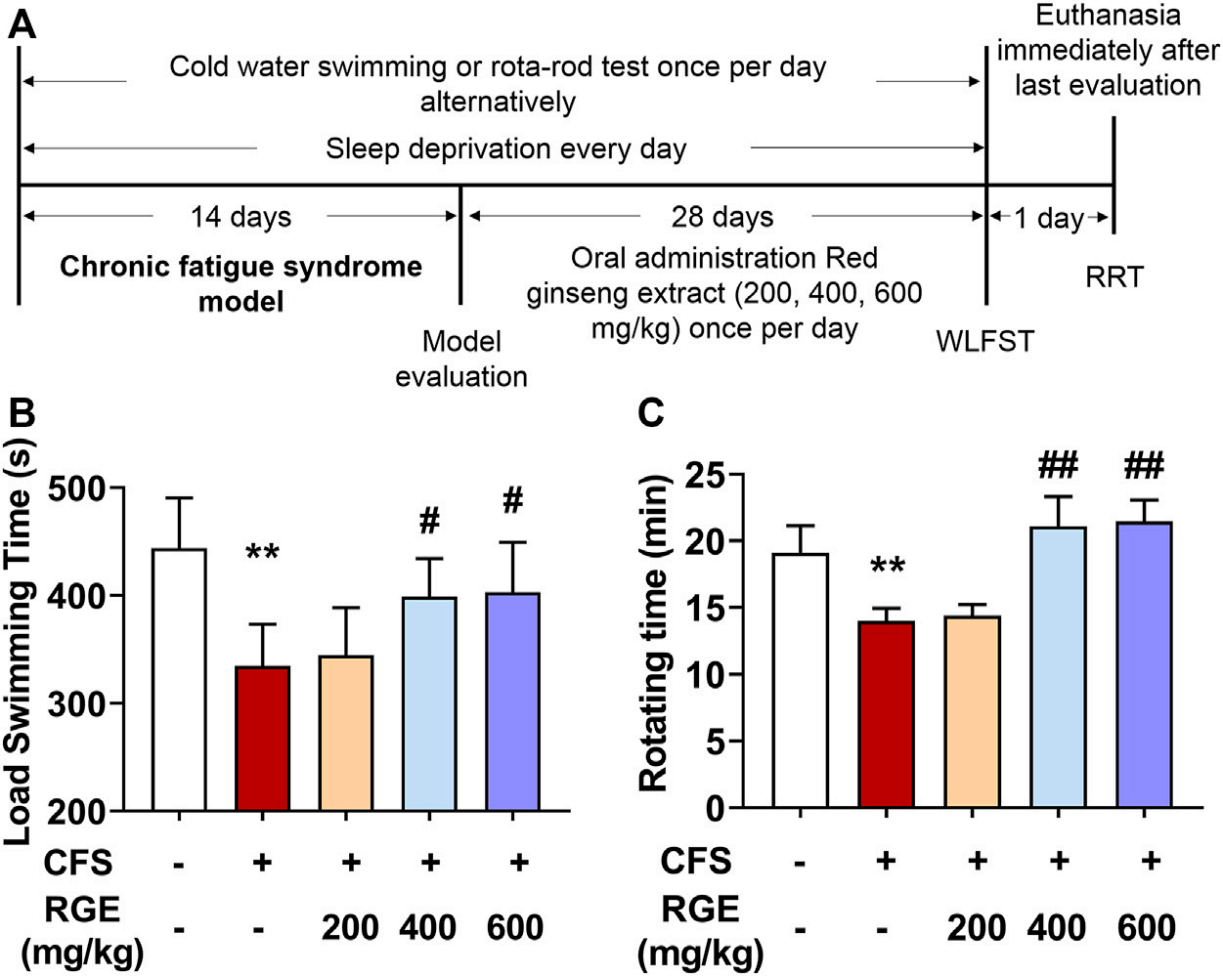
The effects of red ginseng extract (RGE) on weight-loaded forced swimming test (WLFST) and rota-rod test (RRT) in multiple stress factors-induced chronic fatigue syndrome mice. **(A)**. Experiment scheme of the study. **(B,C)**. Load swimming time **(B)** and rotating time **(C)** were recorded in the WLFST and RRT after the last administration of RGE or vehicle at 44th or 46th day. Mice from control or chronic fatigue syndrome model group were treated with water or different dose of RGE (200, 400 or 600 mg/kg). n = 13 mice per group. Compared with control group, ^**^ *p* < 0.01; compared with CFS group, ^#^ *p* < 0.05, ^##^ *p* < 0.01.

WLFST, the most widely used and objective index to evaluate the anti-fatigue effect of drugs, was used in these studies while the rotating experiment was used to test the balance and neuromuscular coordination of mice (Sun et al., 2018). As shown in Figure 1, the load swimming time and rotating time of CFS model mice dramatically reduced compared with that of the control group, while 400 or 600 mg/kg RGE treatment significantly restored the physical capability of the animals compared with the CFS model group, which suggested that the extracts of red ginseng had a certain anti-fatigue effect.

### 3.2 Red ginseng alleviates skeletal muscle lactate acid (LA), serum lactate dehydrogenase (LDH) and urea concentration in mice with chronic fatigue

LA is the metabolite of pyruvate and hydrogen produced by glycolysis of carbohydrates such as glycogen in the body. With the extension of exercise time, the accumulation of LA in the body affects skeletal muscle contractility and the exercise endurance of the body (Lima et al., 2016; Patikas et al., 2018). LDH is an important enzyme in glycolysis; it catalyzes the redox reaction between pyruvate and lactic acid. Serum urea (urea) is the metabolite of protein and amino acid decomposition in the body and is a common index to evaluate the degree of fatigue after exercise (Hu et al., 2021). LA, LDH and urea were widely used as biochemical indicators for evaluating exercise-induced fatigue (Yan et al., 2022).

**FIGURE 2:**
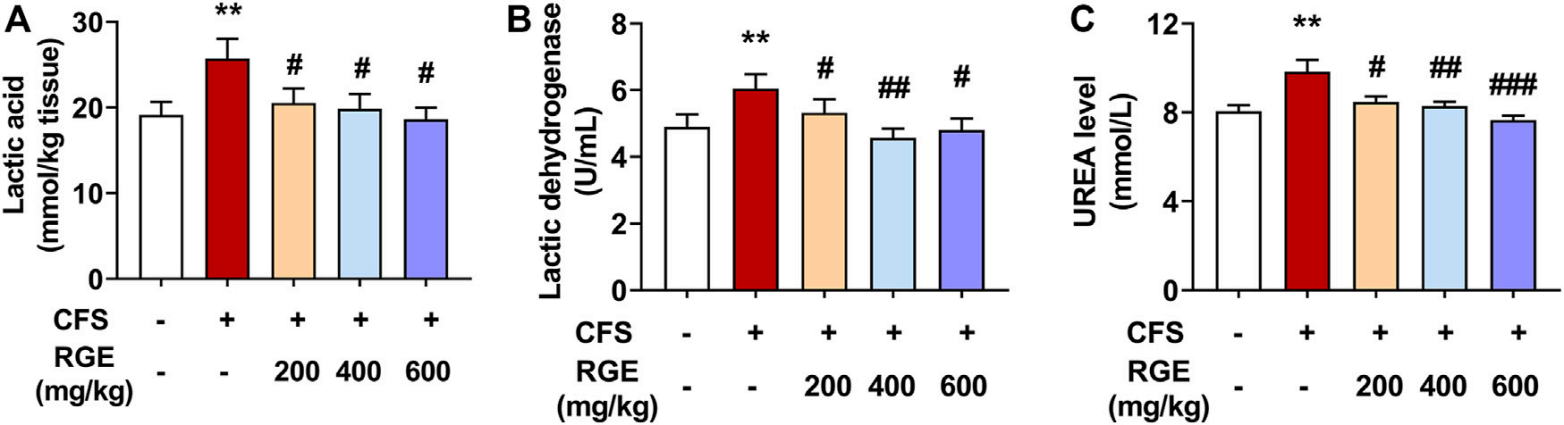
The effects of RGE on lactic acid, lactic dehydration and urea in chronic fatigue syndrome mice. **(A)**. Lactic acid content in tibialis anterior (TA) muscles of the indicated groups. **(B,C)**. Lactic dehydration and urea in serum of the indicated groups. Mice from control or chronic fatigue syndrome model group were treated with water or different dose of RGE (200, 400 or 600 mg/kg). n = 6 mice per group. Compared with control group, ^**^ *p* < 0.01; compared with CFS group, ^#^ *p* < 0.05, ^##^ *p* < 0.01, ^###^ *p* < 0.001.

In this study, compared with control group, exposed to long-term multiple stress factors significantly increased the levels of LA, LDH, and urea in the CFS model group (34% for LA; 23% for LDH; 22% for urea), while administration of red ginseng (200, 400 and 600 mg/kg) for 28 days decreased these indicators in a dose-dependent manner compared with the CFS group (Figures 2A–C). These findings show that RGE improved the energy supply and utilization capacity of the metabolic system, promoted the oxidation and reduction reaction of lactic acid, improved the exercise level of the body, and improved the bodies return to normalcy in mice.

### 3.3 Red ginseng rescues degeneration of mitochondrial morphology in skeletal muscle of fatigued mice

The skeletal muscle is the largest organ and has the highest energy metabolism requirements of the human body. Skeletal muscle is rich in mitochondria, the latter being the key to their metabolic function and physiological or pathological response. Multiple clinical studies have shown that exercise-induced fatigue can lead to a series of mitochondrial metabolic changes in skeletal muscle, which in turn affects exercise ability and health. Recent studies have found that skeletal muscle has a highly dynamic mitochondrial network to meet the needs of muscle contraction and energy metabolism caused by various physiological and pathological stimuli (Gan et al., 2018).

**FIGURE 3:**
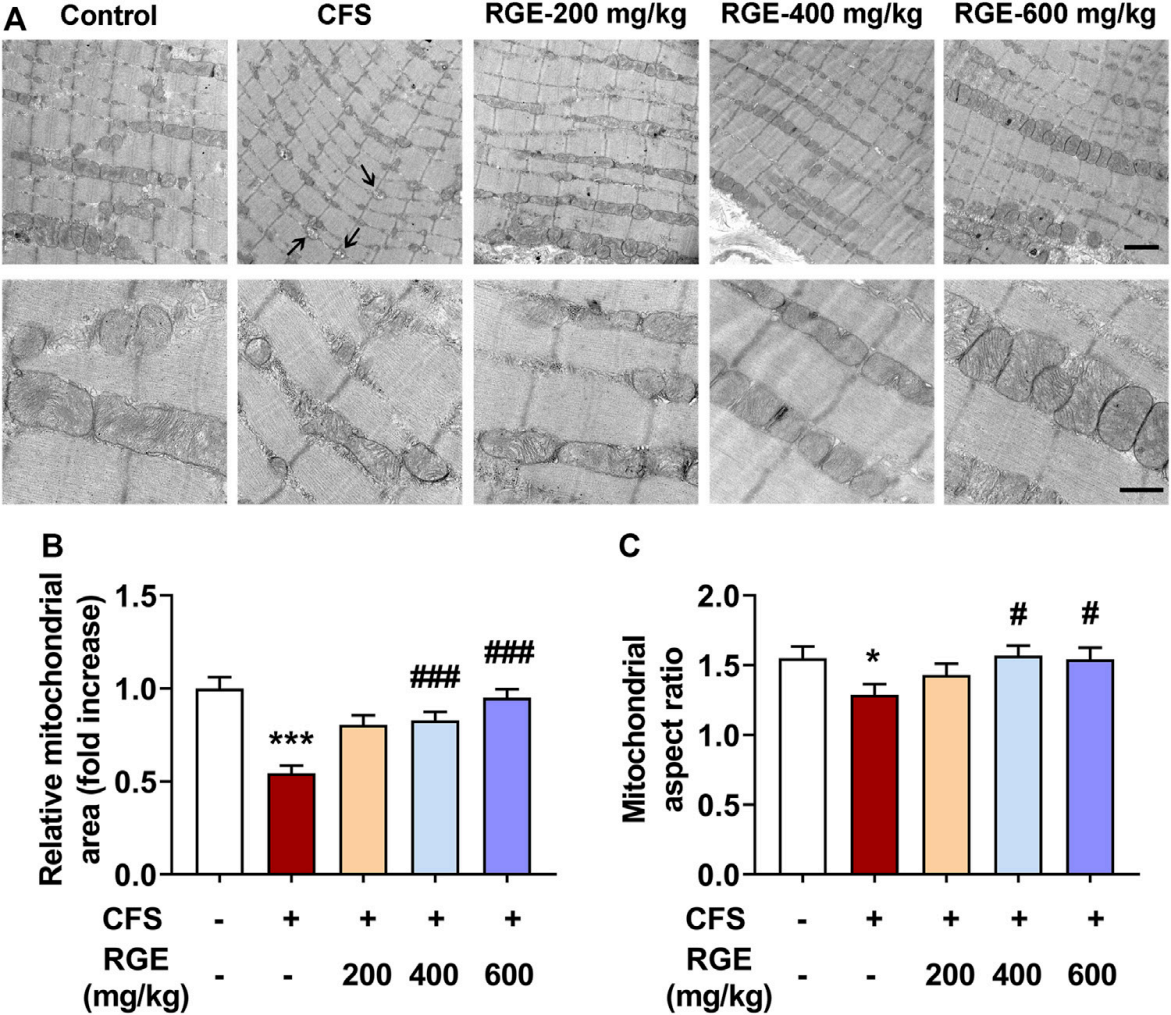
The effect of RGE on mitochondrial morphology in TA muscle of multiple stress factors-induced chronic fatigue mice. **(A)**. Morphology of mitochondria was observed by Transmission Electron Microscopy with magnification of 50,000x or 15,000x. Note the vacuolation degeneration of mitochondria with vague and dissolved cristae (arrows). Scale bar = 2 μm (up panel), or 0.5 μm (down panel). B-C. Quantitative analysis of mitochondrial area **(B)** and aspect ratio **(C)** in TA muscles of the indicated groups. Mice from control or chronic fatigue syndrome model group were treated with water or different dose of RGE (200, 400 or 600 mg/kg). *n* = 13 representative EM fields per group. Compared with control group, ^*^ *p* < 0.05, ^***^ *p* < 0.001; compared with CFS group, ^#^ *p* < 0.05, ^###^ *p* < 0.001.

As depicted in the electron micrographs (Figure 3A), mitochondria from TA muscle of control mice were oval or rounded rectangle with clearly and densely packed cristae structure, while those from mice with CFS were more fragmented round spheres and of low density with occasional vacuolation degeneration accompanied by vague and dissolved cristae (arrows). After treatment with different concentrations of RGE, the density, morphology, and structure of mitochondria normalized. The quantified mitochondrial area and aspect ratio were significantly reduced after long-term multiple chronic fatigue stimulation, while RGE administration alleviated this condition (Figures 3B,C).

### 3.4 Red ginseng ameliorates mitochondrial biogenesis through the AMPK/PGC1α cascade in skeletal muscle of fatigued mice

AMPK is the main switch of energy metabolism. It is an enzyme extremely sensitive to energy change and conversion, which activates the biogenesis effect of mitochondria through phosphorylation (Jager et al., 2007; Morales-Alamo and Calbet, 2016). Activation of PGC-1α promotes transcription of nuclear-encoded mitochondrial genes and regulates mitochondrial biogenesis (Anderson and Prolla, 2009), and its abnormal expression and activity will lead to related metabolic diseases (Rius-Perez et al., 2020). Moreover, PGC-1α effectively improves the quantity and quality of mitochondria in skeletal muscle (Kang et al., 2015; Kang and Ji, 2016), and activates a variety of genes related to oxidative phosphorylation, promotes the oxidative phosphorylation of substrates, drives the synthesis of ATP, and prevents muscle dysfunction (Rowe et al., 2010; Yu et al., 2018).

**FIGURE 4:**
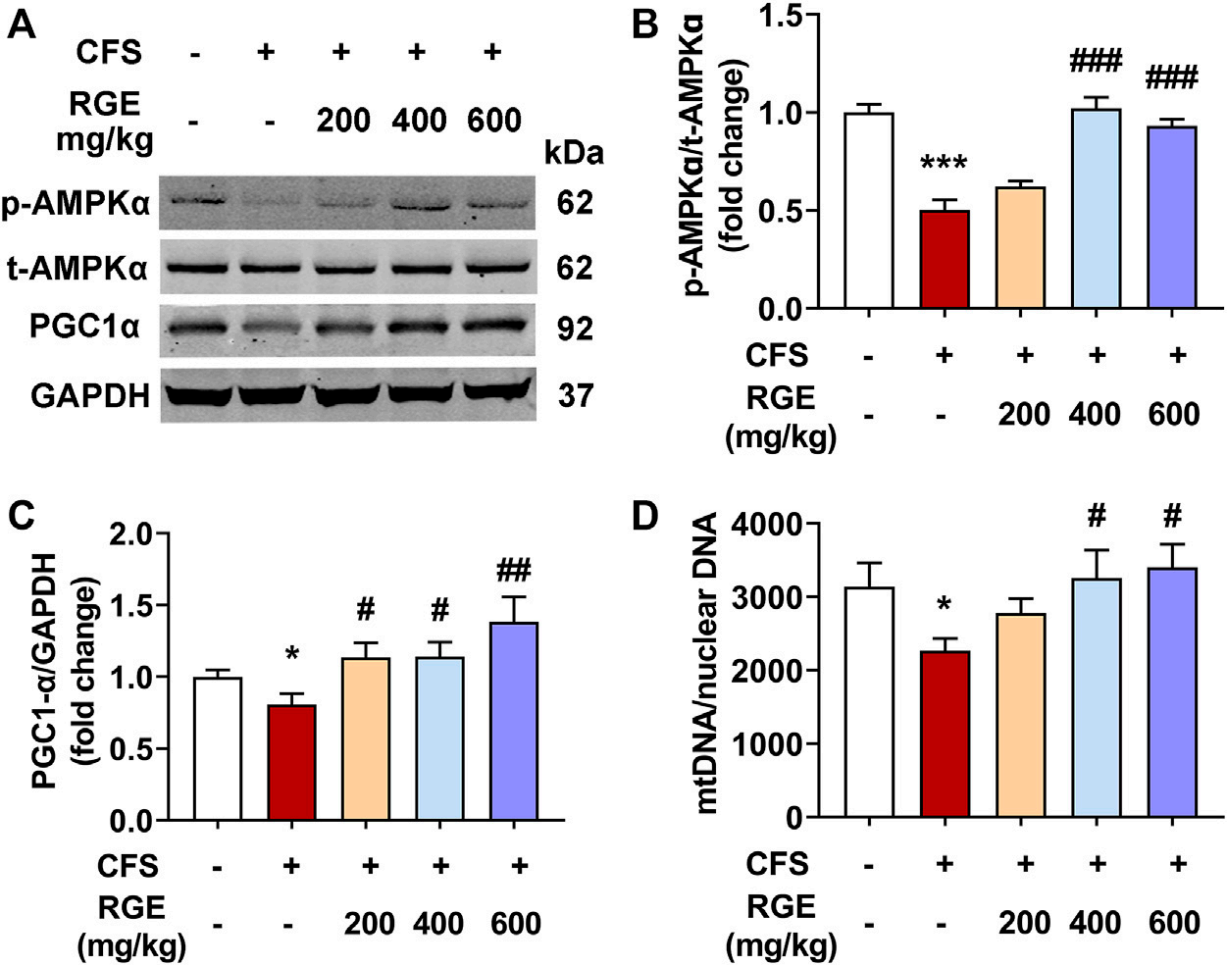
RGE activates AMPK/PGC-1α cascade in TA muscle of multiple stress factors-induced chronic fatigue mice. **(A)**. Representative western blotting images of p-AMPKα, t-AMPKα, PGC1α and GAPDH. **(B,C)**. Relative expression of p-AMPKα, t-AMPKα and PGC1α were quantified. *n* = 3 independent blots. **(D)**. Analysis of mtDNA/nuclear DNA by qPCR, the relative expression of NADH dehydrogenase 1 (mtDNA) to TFAM (nuclear DNA) was quantified using real-time quantitative PCR to determine the relative copy number differences of mtDNA. *n* = 6 per group. Mice from control or chronic fatigue syndrome model group were treated with water or different dose of RGE (200, 400 or 600 mg/kg). Compared with control group, ^*^ *p* < 0.05, ^**^ *p* < 0.01, ^***^ *p* < 0.001; compared with CFS group, ^#^ *p* < 0.05, ^##^ *p* < 0.01, ^###^ *p* < 0.001.

Immunoblot analysis was performed to determine the effect of chronic fatigue stimulation on the phosphorylation level of AMPK and expression of PGC-1α, as well as the therapeutic effect of RGE. As shown in Figure 4, the phosphorylation of AMPK and expression of PGC-1α in skeletal muscle of mice with chronic fatigue were markedly decreased compared with that observed in control mice, while 200, 400, or 600 mg/kg RGE treatment significantly restored the expression of p-AMPK and PGC-1α (Figures 4A–C). This shows that RGE promotes skeletal muscle energy metabolism and ameliorates the progress of fatigue. In addition, the effect of RGE on mitochondrial biogenesis was determined by comparing the ratio of mitochondrial to nuclear DNA copies. This ratio was markedly decreased in mice with chronic fatigue, while 400 and 600 mg/kg RGE administration significantly alleviated this condition (Figure 4D).

### 3.5 Red ginseng attenuates CFS-induced oxidative stress in mice with chronic fatigue

Na^+^-K^+^-ATPase is an enzyme embedded in the lipid bilayer structure of the cell membrane and is closely related to cell ion homeostasis. It drives ion transport inside and outside the membrane, regulates cell osmotic pressure, maintains membrane potential stability, and transmits cell impulse signals (Wirth and Scheibenbogen, 2021), which affects the energy metabolism, internal environment stability, and maintains the integrity of organelles (Fraser et al., 2002). A decrease in Na^+^-K^+^-ATPase activity will increase the content of ROS in mitochondria, damage mitochondrial function, increase concentration of Na^+^ in cells, reduce energy synthesis, and induce fatigue (Hostrup et al., 2017). Mitochondria produce abnormal endogenous ROS when normal physiological functions are disrupted, and many researchers believe that ROS free radical damage plays an important role in the occurrence of fatigue (Muluye et al., 2016).

**FIGURE 5:**
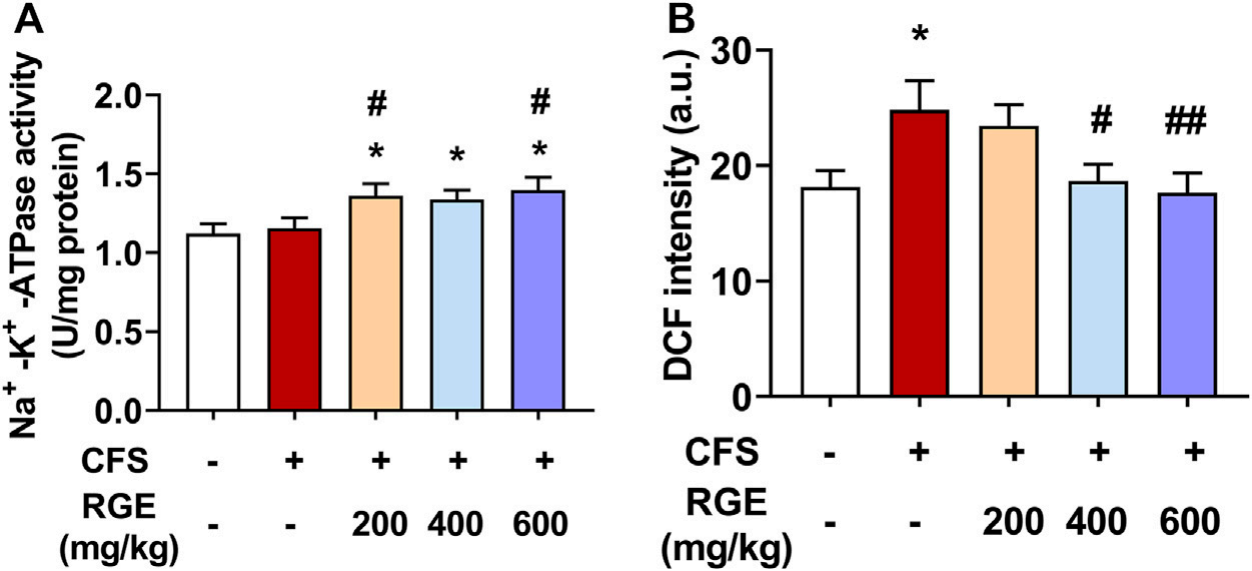
The effects of RGE on skeletal muscle Na^+^-K^+^-ATPase activity and muscle mitochondrial ROS (DCF intensity) in chronic fatigue syndrome mice. **(A–B)**: Na^+^-K^+^-ATPase activity **(A)** and mitochondrial ROS **(B)** in TA muscles of the indicated groups. Mice from control or chronic fatigue syndrome model group were treated with water or different dose of RGE (200, 400 or 600 mg/kg). n = 6 per group. Compared with control group, ^*^ *p* < 0.05, ^**^ *p* < 0.01; compared with CFS group, ^#^ *p* < 0.05, ^##^ *p* < 0.01.

In this study, Na^+^-K^+^-ATPase activity was higher in skeletal muscle of 200, 400 or 600 mg/kg RGE-treated CFS mice than in controls or models (Figure 5A). Interestingly, exposure to long-term multiple stress factors did not affect Na^+^-K^+^-ATPase activity. Consistently, DCF intensity, which indicates the level of ROS, was significantly increased by 37% in skeletal muscle mitochondria of mice with CFS (*p* < 0.05; Figure 5B). However, RGE (400 and 600 mg/kg) treatment showed a dose-dependent decrease in DCF intensity compared to the CFS model group (*p* < 0.05, *p* < 0.01).

### 3.6 Red ginseng protects against CFS-induced mitochondrial dysfunction in skeletal muscle of fatigued mice

Mitochondria are the main regions for the biological oxidation of the three nutrients required to transform energy (tangible and intangible) for muscle contraction, nerve impulse conduction, molecular and ion transport, as well as cell differentiation and proliferation. To determine whether RGE could ameliorate normal function of mitochondria, CcO activity, mitochondrial membrane potential, ATP levels and expression of ACO2 and complex I were assessed.

**FIGURE 6:**
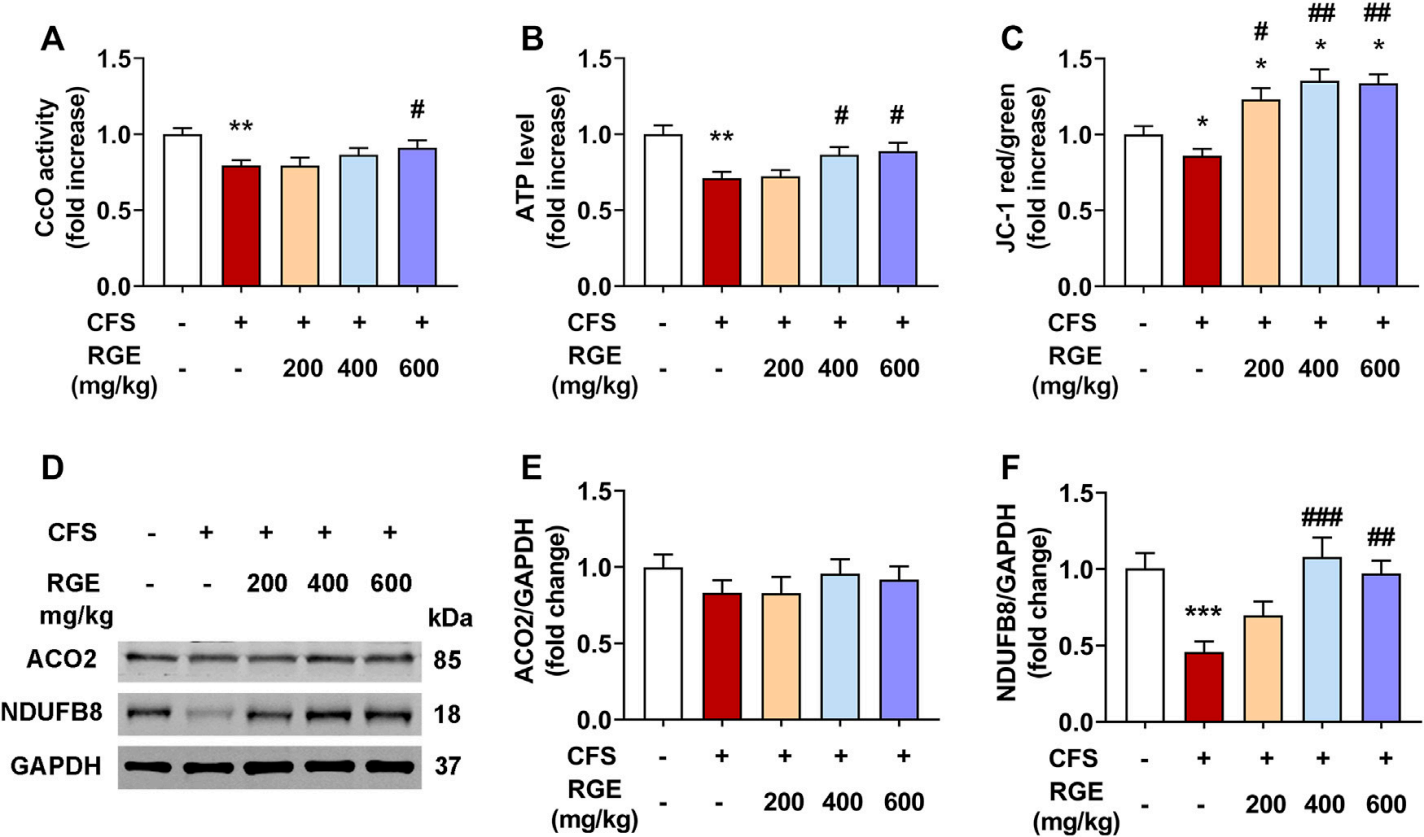
The effects of RGE on mitochondrial function-related indexes in chronic fatigue syndrome mice. **(A–C)**. Cytochrome c oxidase (CcO) activity **(A)**, ATP level **(B)** and mitochondrial membrane potential (JC-1 red/green, **(C)** in TA muscles of the indicated groups. **(D)**. Representative western blotting images of ACO2, respiratory chain complex I (NDUFB8) and GAPDH. **(E,F)**. Relative expression of ACO2 and complex I (NDUFB8) were quantified. *n* = 3 independent blots. Mice from control or chronic fatigue syndrome model group were treated with water or different dose of RGE (200, 400 or 600 mg/kg). *n* = 6 per group. Compared with control group, ^*^ *p* < 0.05, ^**^ *p* < 0.01, ^***^ *p* < 0.001; compared with CFS group, ^#^ *p* < 0.05, ^##^ *p* < 0.01, ^###^ *p* < 0.001.

As shown in Figures 6A,B, the CcO activity and ATP levels in mice with CFS were significantly decreased (∼by 20%, 29%; both *p* < 0.01) compared with the control group, while 400 or 600 mg/kg red ginseng administration markedly reversed this pattern. Subsequently, mitochondrial membrane potential was determined by comparing the ratio of red to green fluorescence in each group. Compared with the control group (Figure 6C), JC-1 red/green decreased significantly in the skeletal muscle mitochondria of mice with CFS (∼by 14%, *p* < 0.01), indicating a loss of △ΨM, which was alleviated by administration of 200, 400, or 600 mg/kg red ginseng (∼by 23%, 35%, 34%; *p* < 0.05, *p* < 0.01, *p* < 0.01). In addition, the expression of respiratory chain complex I (NDUFB8) were dramatically decreased (∼by 54%, *p* < 0.001) in mice with CFS, while the long-term multiple chronic fatigue stimulation did not obviously affect ACO2 expression (Figure 6D–F). After treatment with different concentrations of RGE, the expression of complex I tended to be normal.

## 4 Discussion

Chronic fatigue involves the inability of the body to sustain a normal level of physiology function, or to maintain prolonged common exercise intensity. Therefore, early studies viewed fatigue as the main inducing factor of the condition and not a pathological factor. However, if effective measures are not in place to actively eliminate fatigue caused by sports training or physical fitness, it will not only affect physical fitness, but also lead to pathological changes that are detrimental to health (Cordeiro et al., 2017; Proschinger and Freese, 2019).

Traditional Chinese medicine mostly classifies fatigue as “Qi Deficiency” syndrome of “virtual labour”, “excessive fatigue” and “fatigue leads to Qi consumption”. Some researchers intervened in sports fatigue from the deficiency of Qi, Yang and Zang Fu organs, and generally did not deviate from the idea of “deficiency makes up for it”. In recent years, a large number of “Qi tonifying " drugs have been used in anti-fatigue research, such as ginseng, red ginseng, and *Astragalus* membranaceus. Among these, ginseng is one of the most widely used herb recorded in pharmacopoeia of China, Russia, Korea, Japan, United States and European Union (Shikov et al., 2021; Li et al., 2022). And *P. ginseng* has been well established as an adaptogen which comprises of plant extract or natural compounds that promote adaptability and survival of living organisms in stress (Panossian et al., 2021). Studies suggested that the water extract of red ginseng can prolong the weight-bearing swimming time of fatigued mice, increase the accumulation of liver glycogen, reduce the content of serum lactic acid, enhance the adaptability of the body to exercise load through antioxidation, and prevent fatigue (Min et al., 2003; Zhang and Guo, 2021).

In modern medicine, it is believed that physical and mental exhaustion, accumulation of metabolites, and an imbalance of the internal environment leads to fatigue related to disorders in material and energy metabolism. The external performance associated with body fatigue can be measured using the exhaustion time test. The body mainly obtains energy through glycogen fermentation during exercise; therefore, glycogen consumption leads to a decline in the body’s activity capacity, glycolysis, and the accumulation of its metabolite LA, causing fatigue (Wan et al., 2017). As the intensity of exercise load increases, the body consumes a lot of protein and produces a lot of metabolites, which leads to an increase in blood urea (Tao and Zou, 2021). LDH is an important enzyme in the process of glucose metabolism that catalyses the reversible reaction between acetone and LA. It can thus enhance the metabolic reaction of LA and provide energy for the body (Wang et al., 2015). Na^+^-K^+^-ATPase is an important protease on cell membranes which can affect energy metabolism, internal environment stability, and maintain organelle integrity. Na^+^-K^+^-ATPase hydrolyses ATP, releases energy, maintains the gradient difference of Na^+^ and K^+^ ions inside and outside cells, and maintains cell homeostasis (Castillo et al., 2015). The decrease in Na^+^-K^+^-ATPase activity increases oxygen free radicals in mitochondria, destroys the function of mitochondria, increases the concentration of Na^+^ in cells, and reduces the level of energy synthesis and fatigue (Sostaric et al., 2022). Skeletal muscles have high energy requirements, especially during intense exercise (Johnson et al., 2013). Owing to this, adequate mitochondrial function is vital to the function of skeletal muscle cells. Previous studies showed that excessive intense exercise impairs enzymes of the mitochondrial respiratory chain, causing decreased ATP levels and △ΨM, as well as increased calcium influx, ROS production, and cytochrome c leakage (Shi et al., 1999; Jose et al., 2013; Filler et al., 2014; Arribat et al., 2019; Hood et al., 2019). These classical indicators are widely used to evaluate the physical condition of the human body in clinical tests. The current study findings show that RGE can prolong the time of forced swimming and rotating rod in mice with chronic fatigue, reduce LA, serum LDH, and urea in skeletal muscle, increase the activity of Na^+^-K^+^-ATPase in skeletal muscle, and increase the levels of CcO and ATP. This shows that RGE can improve the body’s glycogen reserve, promote the clearance of glucose metabolites, promote ATP hydrolysis, provide energy for skeletal muscle cells, improve mitochondrial function, maintain the stability of ions inside and outside cells, and reduce damage to skeletal muscle cells.

AMPK is an enzyme that is extremely sensitive to energy changes and conversion; it activates the synthetic effect of mitochondria through phosphorylation (Morales-Alamo and Calbet, 2016). PGC-1α plays an important role in mitochondrial biosynthesis, glucose metabolism, and lipid metabolism (Kang and Ji, 2016). The transcriptional coactivator PGC-1α is known to be the master regulator in the biogenesis of mitochondrion because it regulates transcription of nuclear-encoded mitochondrial genes and plays an important role in energy metabolism. Owing to this, it is associated with several mitochondrial diseases, such as amyotrophic lateral sclerosis, Huntington’s Disease, and Parkinson’s Disease (Fernandez-Marcos and Auwerx, 2011; Safdar et al., 2016; Islam et al., 2018; Cao et al., 2020). In addition, several studies showed that PGC-1α exhibits anti-fatigue activity in people with cancer-related fatigue and deficiency syndrome (Kim et al., 2020; Yang et al., 2022), which suggests an improvement in the mitochondrial function of skeletal muscle cells.

AMPK acts as the upstream regulator of PGC-1α by regulating the transcriptional activity of PGC-1α to promote the biogenesis of mitochondria and provide energy for tissues and cells (Kim et al., 2018; Yu et al., 2018; Zhang and Liang, 2019). Herein, mice in the model group that underwent multiple stress factors stimulation exhibited decreased expression of p-AMPK and PGC-1α in their skeletal muscles, which is consistent with previous research (Sriwijitkamol et al., 2006; Sanchez-Roige et al., 2014; Dong et al., 2018). Therefore, the improvement in mitochondrial function from RGE administration is closely associated with the activation of the AMPK/PGC-1α pathway, which in turn promotes skeletal muscle energy generation and delays fatigue.

In addition, oral administration with 400 and 600 mg/kg RGE showed quite similar effect in most of the indicators tested in this study, such as load swimming time, rotating time, LDH, mitochondrial aspect ratio, mtDNA/nuclear DNA, Na^+^-K^+^-ATPase activity, ATP level, ATP level and △ΨM, there could be following reasons through analysis: Firstly, the improvement effect of 400 mg/kg RGE might reach or approach the maximal effect of RGE on the current CFS model mice; Secondly, chronic fatigue is complicated physiological phenomenon involves not only skeletal muscle, but also multiple tissues and organs, even psychological impact, while the improvement effect of RGE displayed in various aspect according to numerous literatures (Lee et al., 2015; So et al., 2018; Park et al., 2021; Yoon et al., 2021; Zhang and Guo, 2021; Yang et al., 2022); Thirdly, different from a variety of diseases, the physiological, pathological or biochemical changes of the current CFS model were not very serious, attributed to this was a long-term chronic syndrome, and thus caused the limited dose-dependency of RGE. The action patterns and overall therapeutic mechanisms of red ginseng on CFS still need further investigation.

In conclusion, the anti-fatigue mechanism of red ginseng against multiple stress factors-induced CFS is related to its effective mitochondrial improvement activity, including reducing mitochondrial swelling and mitochondrial membrane permeability, and improving mitochondrial dysfunction by activating the AMPK/PGC-1α cascade pathway.

## Data Availability

The original contributions presented in the study are included in the article/Supplementary Material, further inquiries can be directed to the corresponding authors.

## Abbreviations

AMPK, adenosine 5‘-monophosphate -activated protein kinase; PGC-1α, peroxisome proliferator-activated receptor-γ coactivator-1α; CFS, chronic fatigue syndrome; RGE, red ginseng extract; HPLC, high performance liquid chromatography; RRT, rota-rod test; WLFST, weight-loaded forced swimming test; TA, tibialis anterior; LA, lactate acid; LDH, lactic acid dehydration; mtDNA, mitochondrial DNA; CcO, cytochrome c oxidase; ROS, reactive oxygen species; DCF, dichlorofluorescein; △ΨM, mitochondrial membrane potential

## References

[B1] Anderson G. Maes M. (2020). Mitochondria and immunity in chronic fatigue syndrome. Prog. Neuropsychopharmacol. Biol. Psychiatry 103, 109976. 10.1016/j.pnpbp.2020.109976 32470498

[B2] Anderson R. Prolla T. (2009). PGC-1alpha in aging and anti-aging interventions. Biochim. Biophys. Acta 1790 (10), 1059–1066. 10.1016/j.bbagen.2009.04.005 19371772 PMC2743759

[B3] Arribat Y. Broskey N. T. Greggio C. Boutant M. Conde Alonso S. Kulkarni S. S. (2019). Distinct patterns of skeletal muscle mitochondria fusion, fission and mitophagy upon duration of exercise training. Acta Physiol. (Oxf) 225 (2), e13179. 10.1111/apha.13179 30144291

[B4] Cao K. Lv W. Hu S. Gao J. Liu J. Feng Z. (2020). Punicalagin activates AMPK/PGC-1α/Nrf2 cascade in mice: The potential protective effect against prenatal stress. Mol. Nutr. Food Res. 64 (14), e2000312. 10.1002/mnfr.202000312 32475051

[B5] Castillo J. P. Rui H. Basilio D. Das A. Roux B. Latorre R. (2015). Mechanism of potassium ion uptake by the Na(+)/K(+)-ATPase. Nat. Commun. 6, 7622. 10.1038/ncomms8622 26205423 PMC4515779

[B6] Cordeiro L. M. S. Rabelo P. C. R. Moraes M. M. Teixeira-CoelhoF. Coimbra C. C. Wanner S. P. (2017). Physical exercise-induced fatigue: The role of serotonergic and dopaminergic systems. Braz J. Med. Biol. Res. 50 (12), e6432. 10.1590/1414-431X20176432 29069229 PMC5649871

[B7] Dong J. Z. Wei Y. T. Xu H. Y. Zhang Y. Yong R. L. Xue Y. N. (2018). Electroacupuncture of "zusanli" (ST 36) raises muscular force by adjusting AMPK/PGC-1 alpha signaling in rats with chronic fatigue syndrome. Zhen Ci Yan Jiu 43 (6), 335–340. 10.13702/j.1000-0607.171010 30091537

[B8] Fernandez-Marcos P. J. Auwerx J. (2011). Regulation of PGC-1α, a nodal regulator of mitochondrial biogenesis. Am. J. Clin. Nutr. 93 (4), 884S–890S. 10.3945/ajcn.110.001917 21289221 PMC3057551

[B9] Filler K. Lyon D. Bennett J. McCain N. Elswick R. Lukkahatai N. (2014). Association of mitochondrial dysfunction and fatigue: A review of the literature. BBA Clin. 1, 12–23. 10.1016/j.bbacli.2014.04.001 25147756 PMC4136529

[B10] Fraser S. F. Li J. L. Carey M. F. Wang X. N. Sangkabutra T. Sostaric S. (2002). Fatigue depresses maximal *in vitro* skeletal muscle Na(+)-K(+)-ATPase activity in untrained and trained individuals. J. Appl. Physiol. (1985) 93 (5), 1650–1659. 10.1152/japplphysiol.01247.2001 12381750

[B11] Gan Z. Fu T. Kelly D. P. Vega R. B. (2018). Skeletal muscle mitochondrial remodeling in exercise and diseases. Cell Res. 28 (10), 969–980. 10.1038/s41422-018-0078-7 30108290 PMC6170448

[B12] Hood D. A. Memme J. M. Oliveira A. N. Triolo M. (2019). Maintenance of skeletal muscle mitochondria in health, exercise, and aging. Annu. Rev. Physiol. 81, 19–41. 10.1146/annurev-physiol-020518-114310 30216742

[B13] Hostrup M. JeSSen S. Onslev J. Clausen T. Porsbjerg C. (2017). Two-week inhalation of budesonide increases muscle Na, K ATPase content but not endurance in response to terbutaline in men. Scand. J. Med. Sci. Sports 27 (7), 684–691. 10.1111/sms.12677 27060857

[B14] Hou Y. Tang Y. Wang X. Ai X. Wang H. Li X. (2020). Rhodiola Crenulata ameliorates exhaustive exercise-induced fatigue in mice by suppressing mitophagy in skeletal muscle. Exp. Ther. Med. 20 (4), 3161–3173. 10.3892/etm.2020.9072 32855685 PMC7444336

[B15] Hu G. Gao S. Mou D. (2021). Water and alcohol extracts from Diaphragma juglandis on anti-fatigue and antioxidative effects *in vitro* and vivo. J. Sci. Food Agric. 101 (8), 3132–3139. 10.1002/jsfa.10942 33185274

[B16] Hyun S. H. Kim S. W. Seo H. W. Youn S. H. Kyung J. S. Lee Y. Y. (2020). Physiological and pharmacological features of the non-saponin components in Korean Red Ginseng. J. Ginseng Res. 44 (4), 527–537. 10.1016/j.jgr.2020.01.005 32617032 PMC7322739

[B17] Islam H. Edgett B. A. Gurd B. J. (2018). Coordination of mitochondrial biogenesis by PGC-1α in human skeletal muscle: A re-evaluation. Metabolism 79, 42–51. 10.1016/j.metabol.2017.11.001 29126696

[B18] Jager S. Handschin C. St-Pierre J. Spiegelman B. M. (2007). AMP-activated protein kinase (AMPK) action in skeletal muscle via direct phosphorylation of PGC-1alpha. Proc. Natl. Acad. Sci. U. S. A. 104 (29), 12017–12022. 10.1073/pnas.0705070104 17609368 PMC1924552

[B19] Johnson M. L. Robinson M. M. Nair K. S. (2013). Skeletal muscle aging and the mitochondrion. Trends Endocrinol. Metab. 24 (5), 247–256. 10.1016/j.tem.2012.12.003 23375520 PMC3641176

[B20] Jose C. Melser S. Benard G. Rossignol R. (2013). Mitoplasticity: Adaptation biology of the mitochondrion to the cellular redox state in physiology and carcinogenesis. Antioxid. Redox Signal 18 (7), 808–849. 10.1089/ars.2011.4357 22989324

[B21] Kang C. Goodman C. A. Hornberger T. A. Ji L. L. (2015). PGC-1α overexpression by *in vivo* transfection attenuates mitochondrial deterioration of skeletal muscle caused by immobilization. FASEB J. 29 (10), 4092–4106. 10.1096/fj.14-266619 26178167 PMC4566942

[B22] Kang C. Ji L. L. (2016). PGC-1α overexpression via local transfection attenuates mitophagy pathway in muscle disuse atrophy. Free Radic. Biol. Med. 93, 32–40. 10.1016/j.freeradbiomed.2015.12.032 26746585

[B23] Kim J. W. Han S. W. Cho J. Y. Chung I. J. Lee K. H. Kim J. G. (2020). Korean red ginseng for cancer-related fatigue in colorectal cancer patients with chemotherapy: A randomised phase III trial. Eur. J. Cancer 130, 51–62. 10.1016/j.ejca.2020.02.018 32172198

[B24] Kim M. B. Kim T. Kim C. Hwang J. K. (2018). Standardized kaempferia parviflora extract enhances exercise performance through activation of mitochondrial biogenesis. J. Med. Food 21 (1), 30–38. 10.1089/jmf.2017.3989 29125913

[B25] Lee S. M. Bae B. S. Park H. W. Ahn N. G. Cho B. G. Cho Y. L. (2015). Characterization of Korean red ginseng (panax ginseng meyer): History, preparation method, and chemical composition. J. Ginseng Res. 39 (4), 384–391. 10.1016/j.jgr.2015.04.009 26869832 PMC4593794

[B26] Li H. Jiang H. Xu L. Deng Y. Xu J. Zhao Y. (2022). Effects of different extraction methods in pharmacopoeia on the content and structure transformation of ginsenosides. Molecules 27 (14), 4347. 10.3390/molecules27144347 35889220 PMC9351678

[B27] Lima F. D. Stamm D. N. Della Pace I. D. Ribeiro L. R. Rambo L. M. Bresciani G. (2016). Ibuprofen intake increases exercise time to exhaustion: A possible role for preventing exercise-induced fatigue. Scand. J. Med. Sci. Sports 26 (10), 1160–1170. 10.1111/sms.12549 26589249

[B28] Liu D. Fan H. Wang Y. (2021). Lactobacillus fermentum CQPC08 attenuates exercise-induced fatigue in mice through its antioxidant effects and effective intervention of galactooligosaccharide. Drug Des. Devel Ther. 15, 5151–5164. 10.2147/DDDT.S317456 PMC8714972 34992351

[B29] Liu X. Ming L. Xiangbin L. Lan Z. (2018). Grape seed proanthocyanidin extract supplementation affects exhaustive exercise-induced fatigue in mice. Food Nutr. Res. 62. 10.29219/fnr.v62.1421 PMC5995222 29904333

[B30] McConell G. K. Ng G. P. Y. PhillipsM. Ruan Z. Macaulay S. L. Wadley G. D. (2010). Central role of nitric oxide synthase in AICAR and caffeine-induced mitochondrial biogenesis in L6 myocytes. J. Appl. Physiol. (1985) 108 (3), 589–595. 10.1152/japplphysiol.00377.2009 20044477

[B31] Min Y. K. Chung S. H. Lee J. S. Kim S. S. Shin H. D. Lim B. V. (2003). Red ginseng inhibits exercise-induced increase in 5-hydroxytryptamine synthesis and tryptophan hydroxylase expression in dorsal raphe of rats. J. Pharmacol. Sci. 93 (2), 218–221. 10.1254/jphs.93.218 14578592

[B32] Morales-Alamo D. Calbet J. A. L. (2016). AMPK signaling in skeletal muscle during exercise: Role of reactive oxygen and nitrogen species. Free Radic. Biol. Med. 98, 68–77. 10.1016/j.freeradbiomed.2016.01.012 26804254

[B33] Muluye R. A. Bian Y. Wang L. Alemu P. N. Cui H. Peng X. (2016). Placenta peptide can protect mitochondrial dysfunction through inhibiting ROS and TNF-α generation, by maintaining mitochondrial dynamic network and by increasing IL-6 level during chronic fatigue. Front. Pharmacol. 7, 328. 10.3389/fphar.2016.00328 27729861 PMC5037131

[B34] Natelson B. H. Brunjes D. L. Mancini D. (2021). Chronic fatigue syndrome and cardiovascular disease: JACC state-of-the-art review. J. Am. Coll. Cardiol. 78 (10), 1056–1067. 10.1016/j.jacc.2021.06.045 34474739

[B35] Panossian A. G. Efferth T. Shikov A. N. Pozharitskaya O. N. Kuchta K. Mukherjee P. K. (2021). Evolution of the adaptogenic concept from traditional use to medical systems: Pharmacology of stress- and aging-related diseases. Med. Res. Rev. 41 (1), 630–703. 10.1002/med.21743 33103257 PMC7756641

[B36] Park S. K. Hyun S. H. Park C. K. Kwak Y. S. Jang Y. J. In G. (2021). The antioxidant activities of Korean red ginseng (panax ginseng) and ginsenosides: A systemic review through *in vivo* and clinical trials. J. Ginseng Res. 45 (1), 41–47. 10.1016/j.jgr.2020.09.006 33437155 PMC7790892

[B37] Patikas D. A. Williams C. A. Ratel S. (2018). Exercise-induced fatigue in young people: Advances and future perspectives. Eur. J. Appl. Physiol. 118 (5), 899–910. 10.1007/s00421-018-3823-1 29441401

[B38] Proschinger S. Freese J. (2019). Neuroimmunological and neuroenergetic aspects in exercise-induced fatigue. Exerc Immunol. Rev. 25, 8–19. 30753129

[B39] Rius-Perez S. Torres-Cuevas I. Millan I. Ortega A. L. (2020). PGC-1α, inflammation, and oxidative stress: An integrative view in metabolism. Oxid. Med. Cell Longev. 2020, 1452696. 10.1155/2020/1452696 32215168 PMC7085407

[B40] Rowe G. C. Jiang A. Arany Z. (2010). PGC-1 coactivators in cardiac development and disease. Circ. Res. 107 (7), 825–838. 10.1161/CIRCRESAHA.110.223818 20884884 PMC2955978

[B41] Safdar A. Annis S. Kraytsberg Y. Laverack C. Saleem A. Popadin K. (2016). Amelioration of premature aging in mtDNA mutator mouse by exercise: The interplay of oxidative stress, PGC-1α, p53, and DNA damage. A hypothesis. Curr. Opin. Genet. Dev. 38, 127–132. 10.1016/j.gde.2016.06.011 27497229 PMC5592087

[B42] Sanchez-Roige S. Lalanza J. F. Alvarez-Lopez M. J. Cosin-Tomas M. Grinan-Ferre C. Pallas M. (2014). Long-term wheel running changes on sensorimotor activity and skeletal muscle in male and female mice of accelerated senescence. Age (Dordr) 36 (5), 9697. 10.1007/s11357-014-9697-1 25129573 PMC4159468

[B43] Sandler C. X. Lloyd A. R. (2020). Chronic fatigue syndrome: Progress and possibilities. Med. J. Aust. 212 (9), 428–433. 10.5694/mja2.50553 32248536

[B44] Shi Q. D. Zhang Y. Chen J. Q. Liu S. S. (1999). Electron leak causes proton leak in skeletal muscle mitochondria in exercise-induced fatigue. Sheng Wu Hua Xue Yu Sheng Wu Wu Li Xue Bao (Shanghai) 31 (1), 97–100. 12142925

[B45] Shikov A. N. Pozharitskaya O. N. Makarov V. G. Wagner H. Verpoorte R. Heinrich M. (2021). Medicinal plants of the Russian Pharmacopoeia; their history and applications. J. Ethnopharmacol. 268, 481–536. 10.1016/j.jep.2014.04.007 24742754

[B46] So S. H. Lee J. W. Kim Y. S. Hyun S. H. Han C. K. (2018). Red ginseng monograph. J. Ginseng Res. 42 (4), 549–561. 10.1016/j.jgr.2018.05.002 30337816 PMC6190493

[B47] Sostaric S. Petersen A. C. Goodman C. A. Gong X. Aw T. J. Brown M. J. (2022). Oral digoxin effects on exercise performance, K(+) regulation and skeletal muscle Na(+) , K(+) -ATPase in healthy humans. J. Physiol. 600 (16), 3749–3774. 10.1113/JP283017 35837833 PMC9541254

[B48] Sriwijitkamol A. Ivy J. L. Christ-Roberts C. DeFronzo R. A. Mandarino L. J. Musi N. (2006). LKB1-AMPK signaling in muscle from obese insulin-resistant Zucker rats and effects of training. Am. J. Physiol. Endocrinol. Metab. 290 (5), E925–E932. 10.1152/ajpendo.00429.2005 16352671

[B49] Sun K. Yang P. Zhao R. Bai Y. Guo Z. (2018). Matrine attenuates D-galactose-induced aging-related behavior in mice via inhibition of cellular senescence and oxidative stress. Oxid. Med. Cell Longev. 2018, 7108604. 10.1155/2018/7108604 30598725 PMC6288577

[B50] Tao S. Zou J. (2021). Experimental study on fatigue performance of bushing repair with different fasteners. J. Phys. Conf. Ser. 1885 (3), 032037. 10.1088/1742-6596/1885/3/032037

[B51] Wan J. J. Qin Z. Wang P. Y. Sun Y. Liu X. (2017). Muscle fatigue: General understanding and treatment. Exp. Mol. Med. 49 (10), e384. 10.1038/emm.2017.194 28983090 PMC5668469

[B52] Wang Y. Li Y. Liu Y. Chen X. Wei X. (2015). Extraction, characterization and antioxidant activities of Se-enriched tea polysaccharides. Int. J. Biol. Macromol. 77, 76–84. 10.1016/j.ijbiomac.2015.02.052 25783017

[B53] Wei-Xu F. Zhi-Lai Z. Fang-Jie H. Yu-Guang Z. (2021). Research progress on chemical constituents and pharmacological activities of Ginseng Radix et Rhizoma Rubra. Nat. Prod. Res. Dev. 33 (01), 137–149. 10.16333/j.1001-6880.2021.1.017

[B54] Wirth K. J. Scheibenbogen C. (2021). Pathophysiology of skeletal muscle disturbances in myalgic encephalomyelitis/chronic fatigue syndrome (ME/CFS). J. Transl. Med. 19 (1), 162. 10.1186/s12967-021-02833-2 33882940 PMC8058748

[B55] Yan K. Gao H. Liu X. Zhao Z. Gao B. Zhang L. (2022). Establishment and identification of an animal model of long-term exercise-induced fatigue. Front. Endocrinol. (Lausanne) 13, 915937. 10.3389/fendo.2022.915937 36093084 PMC9459130

[B56] Yang Y. Wang H. Zhang M. Shi M. Yang C. Ni Q. (2022). Safety and antifatigue effect of Korean red ginseng capsule: A randomized, double-blind and placebo-controlled clinical trial. J. Ginseng Res. 46 (4), 543–549. 10.1016/j.jgr.2021.09.001 35818418 PMC9270647

[B57] Yin C. Fu X. Chou J. Li J. Chen Y. Bai J. (2021). A proprietary herbal drug Young Yum Pill ameliorates chronic fatigue syndrome in mice. Phytomedicine 88, 153602. 10.1016/j.phymed.2021.153602 34102522

[B58] Yoon S. J. Kim S. K. Lee N. Y. Choi Y. R. Kim H. S. Gupta H. (2021). Effect of Korean red ginseng on metabolic syndrome. J. Ginseng Res. 45 (3), 380–389. 10.1016/j.jgr.2020.11.002 34025131 PMC8134847

[B59] YuY.ZhaoY.TengF.LiJ.GuanY.XuJ. (2018). Berberine improves cognitive deficiency and muscular dysfunction via activation of the AMPK/SIRT1/PGC-1a pathway in skeletal muscle from naturally aging rats. J. Nutr. Health Aging 22 (6), 710–717. 10.1007/s12603-018-1015-7 29806860

[B60] Zhang F. Guo S. (2021). Immunoregulation effect of red ginseng aqueous extract on mice. Med. Plant 12 (01), 71–74+79.

[B61] Zhang F. Guo S. (2020). Study on anti-fatigue effect and mechanism of aqueous extract of red ginseng. Int. J. traditional Chin. Med. 42 (12), 1132–1136.

[B62] Zhang Q. Liang X. C. (2019). Effects of mitochondrial dysfunction via AMPK/PGC-1 alpha signal pathway on pathogenic mechanism of diabetic peripheral neuropathy and the protective effects of Chinese medicine. Chin. J. Integr. Med. 25 (5), 386–394. 10.1007/s11655-018-2579-0 30656599

[B63] Zhao T. Xie G. Xu L. (2018). Discussion on mechanism of shanghuo caused by Radix ginseng Rubra through AMPK’s function in regulating energy metabolism. J. Zhejiang Chin. Med. Univ. 42 (10), 797–803+809. 10.16466/j.issn1005-5509.2018.10.005

[B64] Zhu H. Xu W. Wang N. Jiang W. Cheng Y. Guo Y. (2021). Anti-fatigue effect of Lepidium meyenii Walp. (Maca) on preventing mitochondria-mediated muscle damage and oxidative stress *in vivo* and vitro. Food Funct. 12 (7), 3132–3141. 10.1039/d1fo00383f 33729250

