# Supplementary material for: Red ginseng extract improves skeletal muscle energy metabolism and mitochondrial function in chronic fatigue mice
Source: Front Pharmacol. 2022 Dec 23;13:1077249. doi: 10.3389/fphar.2022.1077249 (PMC9816794; doi:10.3389/fphar.2022.1077249)
Supplement: Supplementary file 1 [file DataSheet1.PDF]

Supplementary materials

Title: Red ginseng extract improves skeletal muscle energy metabolism and mitochondrial function in chronic fatigue mice

Runing title: RGE improves chronic fatigue

Author names and affiliation: Haijing Zhang [a, 1], Chunhui Zhao [a, 1], Jinli Hou [b,1], Ping Su [a], Yifei Yang [a], Bing Xia [a], Xiaoang Zhao [a], Rong He [a], Lifang Wang [a], Chunyu Cao [a], Ting Liu [a, *] and Jixiang Tian [a, *]

[a] Institute of Chinese Materia Medica, China Academy of Chinese Medical Sciences, Beijing 100700, China;

[b] Experimental Research Center, China Academy of Chinese Medical Sciences, Beijing, 100700, China.

[1] These authors contributed equally to this work.

[*] Corresponding author with complete address: Institute of Chinese Materia Medica, China Academy of Chinese Medical Sciences, No. 16, Dongzhimennei Southern Street, Dongcheng District, Beijing, P.R.China, 100700. Tel: +86 10 65032656. E-mail: ltbit@163.com; jxtian@icmm.ac.cn.

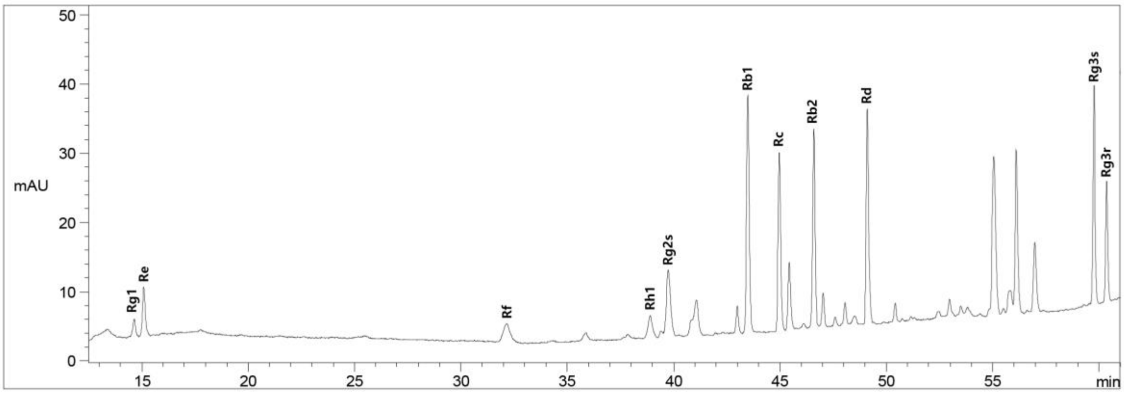

Supplementary Fig. 1. HPLC profile of red ginseng extract (RGE). Ginsenosides from RGE were identified as previously reported [1,2].

Supplementary Table. 1. Contents of 11 ginsenosides in red ginseng extract.

Ginsenoside (mg/g)											
Rg1	Re	Rf	Rh1	Rg2s	Rb1	Rc	Rb2	Rd	Rg3s	Rg3r	Total
0.21	0.71	0.73	0.79	1.09	3.92	2.13	1.79	1.89	2.12	1.12	16.5

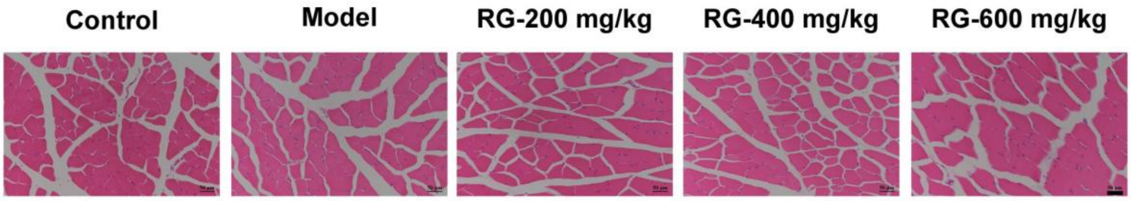

Supplementary Fig. 2. Representative H&E-stained tibialis anterior (TA) muscles of mice from indicated group. Scale bar= 50 μm.

References

1 Shin, S. J. *et al.* Red Ginseng Attenuates Aβ-Induced Mitochondrial Dysfunction and Aβ-mediated Pathology in an Animal Model of Alzheimer's Disease. *Int J Mol Sci* **20**, doi:10.3390/ijms20123030 (2019).

2 Park, H. W. *et al.* Simultaneous determination of 30 ginsenosides in Panax ginseng preparations using ultra performance liquid chromatography. *J Ginseng Res* **37**, 457-467, doi:10.5142/jgr.2013.37.457 (2013).
